# Gene expression profiles of lung adenocarcinoma linked to histopathological grading and survival but not to EGF-R status: a microarray study

**DOI:** 10.1186/1471-2407-10-77

**Published:** 2010-03-02

**Authors:** Jens Neumann, Friedrich Feuerhake, Gian Kayser, Thorsten Wiech, Konrad Aumann, Bernward Passlick, Paul Fisch, Martin Werner, Axel zur Hausen

**Affiliations:** 1 Institute of Pathology, University Hospital Freiburg, Breisacher Str 115a, 79106 Freiburg, Germany; 2 Neuropathology, University Hospital Freiburg, Breisacher Str 115a, 79106 Freiburg, Germany; 3 Department of Thoracic Surgery, University Hospital Freiburg, Hugstetter Straße 55, 79106 Freiburg, Germany

## Abstract

**Background:**

Several different gene expression signatures have been proposed to predict response to therapy and clinical outcome in lung adenocarcinoma. Herein, we investigate if elements of published gene sets can be reproduced in a small dataset, and how gene expression profiles based on limited sample size relate to clinical parameters including histopathological grade and EGFR protein expression.

**Methods:**

Affymetrix Human Genome U133A platform was used to obtain gene expression profiles of 28 pathologically and clinically annotated adenocarcinomas of the lung. EGFR status was determined by fluorescent in situ hybridization and immunohistochemistry.

**Results:**

Using unsupervised clustering algorithms, the predominant gene expression signatures correlated with the histopathological grade but not with EGFR protein expression as detected by immunohistochemistry. In a supervised analysis, the signature of high grade tumors but not of EGFR overexpressing cases showed significant enrichment of gene sets reflecting MAPK activation and other potential signaling cascades downstream of EGFR. Out of four different previously published gene sets that had been linked to prognosis, three showed enrichment in the gene expression signature associated with favorable prognosis.

**Conclusions:**

In this dataset, histopathological tumor grades but not EGFR status were associated with dominant gene expression signatures and gene set enrichment reflecting oncogenic pathway activation, suggesting that high immunohistochemistry EGFR scores may not necessarily be linked to downstream effects that cause major changes in gene expression patterns. Published gene sets showed association with patient survival; however, the small sample size of this study limited the options for a comprehensive validation of previously reported prognostic gene expression signatures.

## Background

Lung cancer is the most common invasive cancer worldwide. In the year 2005 approximately 172.570 new cases were diagnosed in the United States [1]. In addition, it is the leading cause of cancer associated death [2]. Lung cancer includes a broad variety of histological subtypes classified either as small cell lung cancer (SCLC) or non-small cell lung cancer (NSCLC). NSCLC comprises approx. 80% of all lung cancers and is further divided into lung adenocarcinoma (LAC) (~28%), squamous cell carcinoma (SCC) (~44%), and large cell carcinoma (LC) (~9%). However, many tumors are composed of mixed histological types. According to the WHO classification LAC are subdivided into acinar LAC, papillary LAC, bronchioloalveolar carcinoma (BAC), and solid LAC with mucin production as well as a mixed type [2]. The need for diagnostic improvement is underlined by the finding that independent lung pathologists find only 41% agreement on LAC subclassification [3].

Gene expression profiling techniques have led to new approaches to cancer classifications [4]. A number of studies have applied gene expression analyses to identify molecular subgroups of LAC [5-29]. Hierarchical clustering analyses have led to the identification of gene expression profiles associated with patient disease free survival or overall outcome in NSCLC [5,12,16,21,22,25,26,29]. In particular, expression profiles of LAC did not correlate with tumor grade or conventional histopathological subgroups [30,31]. Garber et al. and Bhattacharjee et al. applied unsupervised hierarchical clustering to classify human lung adenocarcinomas [9,14]. Although these studies used different experimental microarray platforms, i.e. oligonucleotide and cDNA microarray, it was surprising to find that both studies show a high congruency in terms of the identified gene signatures [9,14]. Beer et al. could demonstrate that gene expression profiles can be used to calculate a risk index predicting patient survival in early stage LAC [8]. This gene expression signature conferring poor prognosis was independent of stage of disease at time of diagnosis.

A subset analysis of 18 LAC provided by Kikuchi et al. identified an expression signature of 40 genes separating lymph-node positive from lymph-node negative cases [19]. Balko et al. applied gene expression data derived from cell lines showing differential sensitivity to EGFR tyrosine kinase inhibitors to classify human LAC [6].

Other studies also correlated gene expression profiles with prognosis and risk of recurrence [12,16,20,21,25,26,29,32]. In these studies subsets of genes differentially expressed in tumors could predict survival differences among patients with LAC within consistent morphological subgroups. Shedden et al. collected gene expression data and clinical data of 442 LAC from six contributing institutions. In their multi-site blinded validation approach the combination of training-testing methods and clinical data (stage, age and sex) showed the best results in predicting the overall survival [32].

Potti et al. identified gene expression profiles predicting the risk of recurrence in a cohort of 198 NSCLC patients, among them 89 LACs [25]. They computed nine metagene-signatures containing altogether 133 elements using the metagene construction model and binary prediction tree analysis. The metagene-signatures were generated from a training cohort to predict the risk of recurrence and are available online as supplemental information. These signatures have been validated in two multicenter cooperative study group collectives.

A similar study by Larsen et al. provided a 54-gene signature predicting the risk of recurrent disease independently of tumor stage [21]. Both studies point to the potential of gene expression methodologies to refine the accuracy of clinical prognosis for patients undergoing resection for primary LAC especially in early disease stages.

In our study we investigate if elements of published gene sets can be reproduced in a small independent dataset, and how gene expression profiles based on limited sample size relate to clinical parameters including histopathological grade and EGFR protein expression.

## Methods

### Patients and samples

Tissues were selected from the tumor bank of the Institute of Pathology, University Hospital Freiburg. All tissue samples were collected for diagnostic purposes and studied in accordance with national ethical principles. The investigation protocol was approved by the institutional review board (No.14/2004). Clinico-pathological data were collected in collaboration with the Department of Thoracic Surgery, University Hospital Freiburg. Representative 3 μm sections of the tumor tissues were H&E stained and reviewed for tissue quality, cellular composition, confirmation of the histopathological diagnosis and tumor-grading independently by three surgical pathologists (GK, MW, AzH) according to the World Health Organization criteria and current TNM-classification [2]. Discordant cases have been discussed in common and a consensus was defined for the subsequent statistical analyses. Only samples with a tumor-cell content of more than 90% were used for molecular analyses. We analyzed tumor samples of 28 patients who underwent surgery between 2002 and 2004 at the Department of Thoracic Surgery, University Hospital Freiburg. The mean age of the patients was 65.3 years. Forty-eight percent of the patients were male and 52% female. All 28 tumors were initially classified as mixed type adenocarcinomas of the lung. For molecular analyses areas with homogeneous acinar growth pattern were selected. The majority of the tumors was moderately (n = 15) or poorly differentiated (n = 10). The patients were operated with a curative intent. Therefore the operation was performed in early clinical stages (24% at T1-stage and 65.5% at T2-stage). In 83% lymph-node metastases were present at the time of surgery. Only in one case distant metastases could be evaluated. Two cases revealed residual tumor after surgical treatment. Clinico-pathological data are summarized in table 1.

**Table 1:** Clinico-pathological data of 28 LAC patients used for the generation of expression profiles.

Code	Age	Sex	Grade	T	N	M	R	Smoker
LAC_19	58	M	2	2	2	0	0	yes
LAC_20	59	F	3	2	1	0	1	yes
LAC_21	75	M	2	2	0	0	0	yes
LAC_22	60	F	2	1	2	0	0	yes
LAC_23	47	F	3	2	1	0	1	n.a.
LAC_24	61	M	2	4	2	0	0	yes
LAC_25	64	F	3	2	2	1	0	yes
LAC_26	70	M	2	1	0	0	0	yes
LAC_36	72	F	3	2	2	0	0	yes
LAC_37	67	F	2	2	0	0	0	yes
LAC_38	82	M	2	4	2	0	0	yes
LAC_39	69	M	1	2	2	0	0	yes
LAC_40	83	F	1	1	0	0	0	yes
LAC_41	51	F	2	2	0	0	0	yes
LAC_42	78	F	3	2	0	0	0	no
LAC_43	59	F	2	4	2	0	0	yes
LAC_45	69	M	2	2	0	0	0	yes
LAC_47	56	M	2	1	2	0	0	yes
LAC_49	72	M	3	2	2	0	0	yes
LAC_51	65	M	3	2	2	0	0	yes
LAC_52	70	F	2	2	1	0	0	yes
LAC_53	62	F	3	2	1	0	0	yes
LAC_54	54	F	2	1	0	0	0	yes
LAC_55	77	F	2	2	0	0	0	yes
LAC_56	67	M	3	2	0	0	0	yes
LAC_57	73	M	2	1	0	0	0	yes
LAC_58	56	M	3	2	1	X	0	n.a.
LAC_61	76	F	1	2	0	X	0	n.a.

Classification according to WHO and TNM-Classification (6^th^edition 2002). LAC = lung adenocarcinoma; Age = age at operation; F = female; M = male; T = tumor stage; N = absence (0), presence of ipsilateral peribronchial and/or ipsilateral hilar lymph node metastasis (1) or ipsilateral mediastinal and/or subcarinal lymph node metastasis (2); M = absence (0) or presence (1) of distant metastasis; R = absence (0) or presence (1) of residual tumor; X = classification could not be assessed; n.a. = data not available.

### Gene expression profiling

Total RNA was extracted from each frozen tumor specimen using the Qiashredder (Qiagen, Hilden, Germany) and the RNeasy Kit (Qiagen, Hilden, Germany) and biotinylated cRNAs were generated according to the manufacturer's protocol (Affymetrix, Santa Clara, CA). In brief, the biotinylated cRNA was purified using RNeasy affinity columns (Qiagen, Hilden, Germany). RNA quality control was assessed by the 260 nm and 280 nm absorbance ratio and gel electrophoresis. Further sample processing, including labeling, hybridization, and image scanning was performed using the standard Affymetrix protocol. Five μg of total RNA from each tumor specimen, T7-oligo(dT) primers, and Superscript II RT (Invitrogen GmbH, Karlsruhe, Germany) were used for first strand cDNA synthesis. After second strand synthesis, in vitro transcription was performed using Enzo Transcript Labeling Kit (Enzo Life Science, Farmingdale, NY) to generate biotinylated cRNA targets. cRNA targets were fragmented at 94°C for 35 minutes and 15 μg of it was hybridized to HG-U133A chips (Affymetrix Inc, Santa Clara, CA, USA) at 45°C for 16 hours. The arrays were washed and stained with 10 μg/ml streptavidin-phycoerythrin. After signal amplification with biotinylated anti-streptavidin antibodies the arrays were scanned using the GeneChip^® ^Scanner 3000. The HG-U133A chip contains 22.283 probe sets representing 14.564 human genes.

### Data analysis

Following standard data acquisition, the scanned images were quantified according to the Affymetrix GeneChip Manual (Affymetrix Inc., Santa Clara, CA) by using the Data Mining Tool (DMT) 2.0, and Microarray Database software (accessed June 2004) using the Entrez Gene definitions. The probe set IDs were annotated and, for comparison with published gene expression signatures, manually cross-referenced using NetAffx Analysis Center provided by the homepage of the manufacturer (http://www.affymetrix.com, accessed May 2008) [33]. The signals were globally normalized and scaled to a signal intensity of 500. All of the microarrays were examined for surface defects, grid placement, background intensity, housekeeping gene expression (GAPDH and β-Actin), and 3'-/5'- ratio of probe sets. For hybridization control the signals of the controls (BioB, BioC, BioD, and Cre), the scale factors and the background intensities of each array were calculated and compared. The present calls of hybridized microarrays showed a range from 33.2% to 59.9% of all investigated elements (median 55.05%). The mean 3'-/5'- ratio of probe sets for GAPDH and β-Actin was 1.07 (standard deviation: 0.6) and 1.44 (standard deviation: 1.9), respectively. The spike-in controls showed an adequate expression in all cases. The average background signal - generally recommended being less than 100 - varied between 40.88 and 95.38 (median 53.73). The microarray dataset described in this work, including .cel-files, was deposited at the Gene Expression Omnibus under the series accession GSE17475 [34].

Quality controls were performed using Microarray Suite 5.0 software provided by Affymetrix http://www.affymetrix.com according to the manufacturer's recommendations. Data acquisition for gene expression analysis, starting from the .cel files, was performed using the robust multiarray average (RMA) algorithm published by Irizarry et al [35,36] Prior to analysis by supervised and unsupervised clustering algorithms, the RMA-processed dataset was filtered applying a standard variation filter (default) provided by the dChip-Software V1.3 http://www.dchip.org, version 2003. Two-dimensional hierarchical clustering was performed in D-Chip (V1.3, 2003). Statistical analyses were performed using the publicly available R-Software V2.5.0 http://www.r-project.org[37]. Gene Set Enrichment Analysis (GSEA) was performed using publicly accessible software provided by the Broad Institute http://www.broadinstitute.org/gsea/msigdb/downloads.jsp[38,39]. To identify biologically relevant gene sets for GSEA analysis, the search function provided by the website was used to identify curated gene sets (category c2 only) related to EGFR downstream signalling, using EGFR, ERK, MAPK as search terms http://www.broadinstitute.org/gsea/msigdb/collections.jsp#C2. The algorithm computes an enrichment score (ES) that is based on Kolmogorov-Smirnov statistics, provides a nominal p-value, and corrects for multiple testing by calculating the false discovery rate (FDR). A significance level of FDR < 0.05 was accepted. For a detailed mathematical description of the statistical methods, see Ref. [39].

### Tissue microarray

In order to perform standardized immunohistochemical analyses we generated a tissue microarray (TMA) of the 28 primary LAC containing 3 representative cores of each case to account for potential tumor heterogeneity. In cases with variable staining intensities across cores, the mean was recorded.

### Fluorescence in-situ hybridization

Paraffin sections of 5 μm were dewaxed and washed shortly with PBS. As pretreatment the tissue sections were heated in citrate-buffer for 17 minutes and incubated with Pronase E at 37°C for 3 minutes. Denaturation was performed by formamide 70% for 15 minutes at 75°C and afterwards stabilized by ethanol. The sections were then hybridized with the Vysis EGFR/CEP7 Dual Color Probe for 20 hours at 37°C, after washing the probes were counterstained with Dapi.

### EGFR immunohistochemistry

All slides of the TMA were submitted to immunohistochemistry (IHC) at the same time. In brief, 3 μm thick paraffin sections were analyzed for protein expression of non-phosphorylated EGFR by IHC using the Dako EGFR pharm Dx™ kit (Dako, Germany). The staining procedure was performed according to the provided automated staining protocol on a DAKO autostainer. Afterwards the slides were immersed in hematoxylin for 3 minutes for nuclear counterstaining. For scoring of EGFR expression, the following qualitative scale: 0 - "negative", 1 - "weak staining", 2 - "moderate staining", 3 - "strong staining" was applied. Further the percentage of positive tumor cells was calculated. Both scoring systems were applied for membranous and cytoplasmic positivity.

## Results

### Unsupervised Analysis of Microarray Data

**Figure 1:**
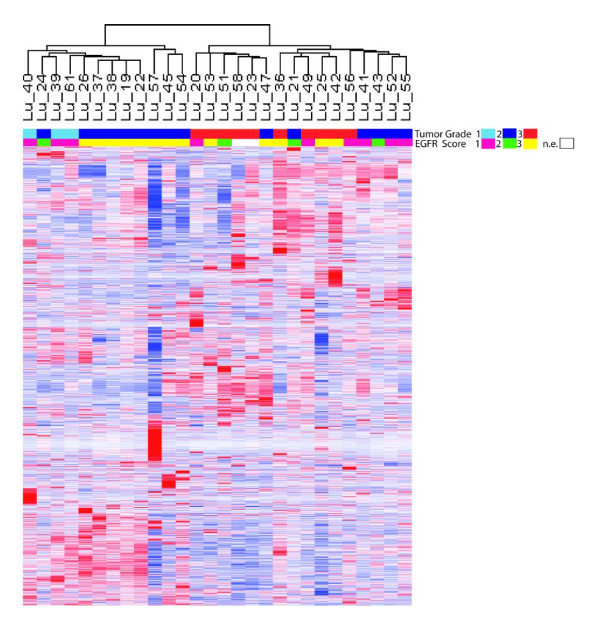
**Hierarchical cluster analysis identifies tumor grade as dominant substructure**. Hierarchical clustering of 2777 features selected by variation filter shows that the dominant substructure of the dataset is driven by tumor grade and not by EGFR status. All grade 3 tumors were segregated in one major branch. No correlation of EGFR status and the results of hierarchical clustering could be obtained. The upper bar shows the histopathological tumor grade (turquoise = grade 1, blue = grade 2 and red = grade 3). The second bar shows the EGFR expression on protein level (pink = no protein expression, green = incomplete membranous stain and yellow = complete membranous stain). The expression values are indicated by color code (blue = no gene expression and red = high gene expression).

After normalization two-dimensional hierarchical clustering analysis was applied to determine if any clinical or biological subset existed in our set of 28 LAC. A final filtered gene list of 2777 probes selected by variation filter provided by dChip-software was used. LACs were clustered into two distinct groups of 16 and 12 samples (fig. 1). The two clusters revealed significant differences with respect to histopathological grading (grade 3 vs. grade 1 and 2; p < 0.001). All well differentiated LAC (G 1; n = 3) were found in cluster 1. In contrast, all poorly differentiated LAC (G 3; n = 10) were present in cluster 2. Although the majority (n = 9) of moderately differentiated LAC (G2) was found in cluster 1, some of these tumors were grouped into cluster 2 (n = 6). No significant association between the two clusters and tumor stage, smoking status, gender, age or immunohistochemical EGFR protein expression was identified. Further, the major clusters obtained by unsupervised analysis did not reflect the clinical outcome with regard to overall survival.

### EGFR immunohistochemistry and FISH analysis in Relation to EGFR mRNA

**Figure 2:**
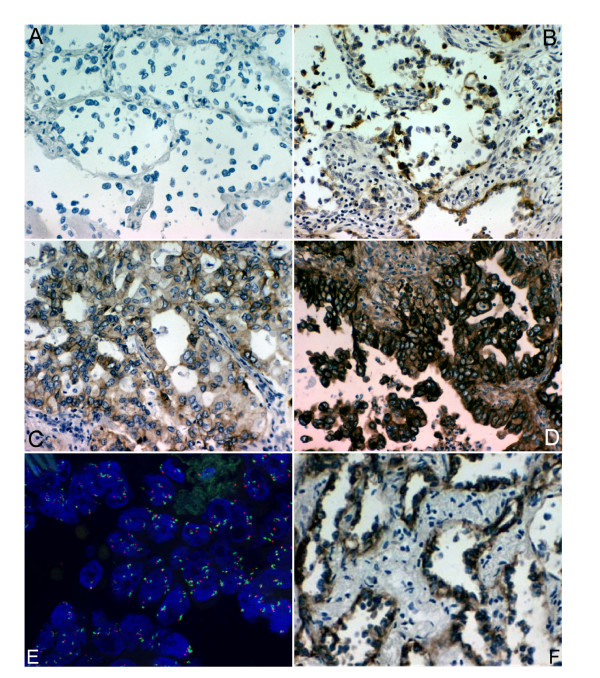
**Quantitative and qualitative assessment of EGFR status**. **A-D: **Immunohistochemical stain for EGFR of primary lung adenocarcinoma (magnification: 200×). A) No membranous or cytoplasmic stain. B) Weak mostly incomplete membranous stain combined with moderate cytoplasmic stain. C) Moderate mostly incomplete membranous stain combined with weak cytoplasmic stain. D) Strong mostly complete membranous stain combined with strong cytoplasmic stain. **E and F: **E) Fluorescence in-situ hybridization for EGFR (locus 7p12, red) and CEP7 (locus 7p11.1-q11.1, green) of primary lung adenocarcinoma showing no amplification of the EGFR-gen. F) Corresponding immunohistochemical stain for EGFR showing strong mostly incomplete membranous staining combined with moderate cytoplasmic staining.

**Figure 3:**
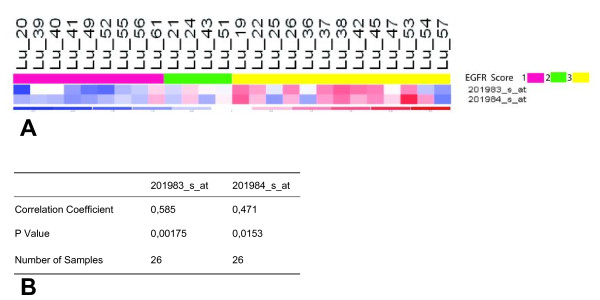
**EGFR expression congruency**. **A: **Comparison of the intensity of positive staining of EGFR obtained by immunohistochemistry and gene expression measured by Affymetrix gene chips: Gene expression values are congruent with EGFR detection on the protein level. The upper bar shows the EGFR expression on protein level (pink = no protein expression, green = incomplete membranous stain and yellow = complete membranous stain). The expression values of elements representing EGFR gene expression (middle) are indicated by color code shown in the lowest bar (blue = low gene expression and red = high gene expression). In **B **the results of the Spearmans Rank Order Correlation of the gene expression values obtained by microarray analyses and EGFR protein levels measured by IHC are shown.

Due to multiple usage of the TMA in two cases the paraffin-embedded material was exhausted. Of the remaining 26 LAC samples nine showed no membranous expression of EGFR. In tumors with positive EGFR-immunohistochemistry 65.38% (+/- 36.35%) of the cells showed membranous and 88.64% (+/- 32.74%) cytoplasmatic staining. Complete membranous staining was seen in 6 cases (23.08%). For these, the intensity score was 3. The incomplete membranous stain was moderate (intensity score 2) in three cases, and strong (intensity score 3) in the remaining eight tumors. The gene expression values obtained by microarray analyses were concordant with EGFR detection on the protein level measured by IHC with p < 0.01 for probe set 201983_s_at, and p < 0.02 for probe set 201984_s_at (Spearmans Rank Order Correlation, see fig. 2 and 3).

*EGFR*-gene amplification was investigated with FISH analysis. On average 3.89 (+/- 1.12) signals for the 7p12 *EGFR *locus and 3.12 (+/- 0.82) CEP 7 signals were detected within the tumor cells. The 7p12/CEP 7 ratio was 1.25 on average. No case revealed *EGFR*-gene locus (7p12/CEP 7 ratio > 2) amplification. No correlation was seen between number of *EGFR*-FISH signals and the intensity of immunohistochemical staining results. Results of the immunohistochemistry and the FISH analysis are shown in fig. 2 and summarized in table 2.

**Table 2:** Results of the EGFR immunohistochemistry and FISH analysis of 26 LAC patients.

	EGFR Immunohistochemistry					EGFR FISH		
				
	Membrane			Cytoplasm				
			
Code	Membrane	Intensity	Percent	Intensity	Percent	Centromere	EGFR Locus	Ratio
Lu_19	complete	3	90	2	90	4.90	6.13	1.25
Lu_20	negative	0	0	0	0	3.94	4.47	1.13
Lu_21	incomplete	2	40	1	60	3.30	3.38	1.02
Lu_22	incomplete	3	90	2	90	2.96	3.31	1.12
Lu_24	complete	2	80	1	90	3.10	3.96	1.28
Lu_25	incomplete	3	20	2	70	3.05	3.44	1.13
Lu_26	complete	3	90	3	90	3.00	4.07	1.36
Lu_36	incomplete	3	70	1	90	3.23	3.99	1.24
Lu_37	incomplete	3	90	2	90	3.14	4.44	1.41
Lu_38	complete	3	70	3	90	4.95	6.13	1.24
Lu_39	negative	0	0	3	40	2.61	3.22	1.24
Lu_40	negative	0	0	1	10	2.35	2.59	1.10
Lu_41	negative	0	0	2	50	3.15	3.84	1.22
Lu_42	incomplete	3	40	3	80	3.55	4.06	1.14
Lu_43	incomplete	2	70	1	80	3.92	6.26	1.60
Lu_45	incomplete	3	60	2	30	2.90	4.12	1.42
Lu_47	complete	3	90	1	90	2.00	2.12	1.06
Lu_49	negative	0	0	0	0	2.87	3.91	1.36
Lu_51	incomplete	2	20	2	40	2.86	3.66	1.28
Lu_52	negative	0	0	1	10	1.93	2.22	1.15
Lu_53	complete	3	80	2	90	2.00	3.31	1.66
Lu_54	incomplete	3	70	2	40	3.96	5.00	1.26
Lu_55	negative	0	0	1	20	2.59	2.45	0.95
Lu_56	negative	0	0	0	0	2.25	4.12	1.83
Lu_57	incomplete	3	80	2	90	2.52	2.54	1.01
Lu_61	negative	0	0	3	80	2.29	2.49	1.09

### Pathway Analysis

**Figure 4:**
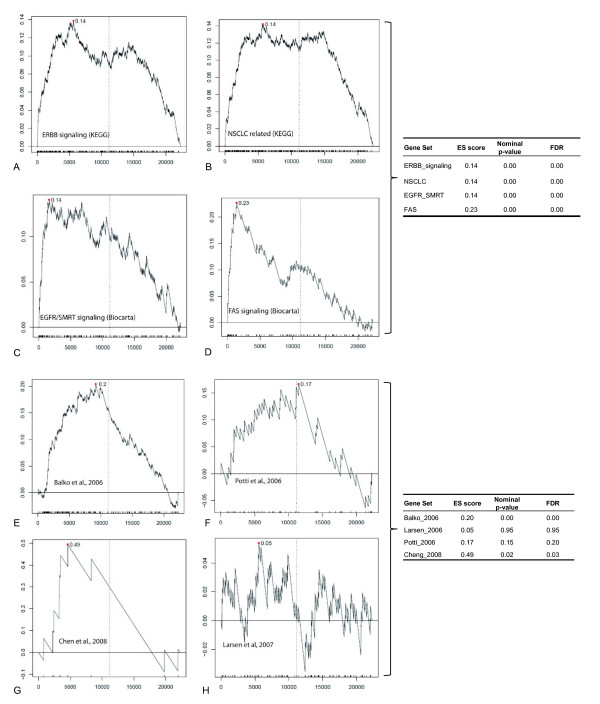
**GSEA Analysis**. **A-D **summarize the results of the pathway-analysis: Four of the gene sets relevant for EGFR-related signaling made available by the GSEA web site (gene set collection "C2") showed statistically significant enrichment towards the top of the data set when it was ranked according to differential gene expression between grade 3 vs. grade 1&2 tumors. **E-H **show the results of GSEA, testing for enrichment of published prognostic gene expression signatures in our small independent data set when it was ranked according to differential gene expression between long-term survivors and patients with unfavorable outcome: The signatures of Balko et al. and Chen et al. were significantly enriched, and the Potti et al. signature showed a clear trend towards enrichment although it did not meet statistical significance. The Larsen signature was not overrepresented at the top of the ranked data set in this analysis. ES = enrichment score, FDR = false discovery rate. A significance level of FDR < 0.05 was accepted.

In a supervised analysis approach, all genes (unfiltered gene set) were ranked according to differential expression in (1) EGFR score 3 vs. EGFR score 0&1&2, (2) EGFR score 2&3 vs. EGFR score 0&1, and (3) histopathological grade 3 vs. grade 2&1. Gene Set Enrichment Analysis (GSEA) was used to test for enrichment of the pathway-related gene sets in the over expressed (top-ranked) or the down regulated (bottom-ranked) genes in each of the supervised analyses. When the dataset was ranked according to differential expression in histopathological grade 3 cases vs. cases classified as grade 2&1, the following gene sets showed significant enrichment after correction for multiple testing: ERBB signaling (KEGG), NSCLC related signaling (KEGG), EGFR/SMRT (Biocarta), and FAS anti-apoptotic signaling (Biocarta). Summarized results are shown in fig. 4A-D. In contrast, no significant enrichment was observed when the dataset was ranked according to EGFR status. For completeness, the other clinical parameters (age, nodal stage etc.) were used to group cases for additional GSEA runs; after correcting for multiple testing none of these analyses showed any statistically significant enrichment of the selected gene sets.

### Assessment of published prognostic gene expression signatures

The published gene signatures were used in two analysis approaches: In an unsupervised analysis, two-dimensional clustering was performed in the space of all genes that were represented on both the published and the U133A platform. Clustering in the space of the "Potti signature", defined as the communality of all genes described as having any prognostic significance (overlap between platforms: 114 probesets, representing 105 Genes, Additional file 1: Supplemental table S1), resulted in co-segregation of cases with similar outcome in two major clusters, one of which included 83% of long-term surviving patients, whereas the other cluster comprised 50% of patients with favorable outcome. Clustering in the space of the other signatures (Larsen 2007, Balko 2006, Chen 2008) did not produce comparable segregation of long term survivors vs. patients with unfavorable outcome [6,12,21]. In a second approach, GSEA was employed to test for enrichment of the signature components at the top of the total data set, ranked according to differential expression of genes between long-term and short-term survival. The gene sets of the Balko, and Chen but not of the Larsen signature showed statistically significant enrichment at the top of data set ranked by survival. The Potti signature showed a trend for enrichment but did not meet statistical significance (see fig. 4). In summary, these results suggest that the association between published prognostic gene expression signatures and outcome is detectable, but does not appear to be a dominant feature of this small independent data set.

## Discussion

In the present study we analyzed 28 LAC for gene expression profile by using the Affymetrix chip platform. Unsupervised hierarchical cluster analysis led to the identification of two subclasses of LAC. We found two clusters revealing significant differences with respect to histopathological grading. All well differentiated LAC (G 1) were found in cluster 1. In contrast, all poorly differentiated LAC (G 3) were present in cluster 2. The interesting finding that some of the moderately differentiated LAC were found in cluster 2, indicates that this subset of moderately differentiated LAC already share gene expression profiles with poorly differentiated LAC, which yet is not reflected by histopathology. Thus, gene expression analysis might help to identify a subgroup of G2-LAC, which already reveals the molecular features of poorly differentiated LAC but lacks typical histomorphological dedifferentiation. These features could be associated with a more aggressive biological behavior of the tumor cells.

The grading system provided by the WHO classification is poorly defined and based on conventional histological criteria, including the extent to which the architectural pattern of the tumor resembles normal lung tissue and cytological atypia. In our study three experienced surgical pathologists reevaluated all cases for confirmation of the diagnosis and tumor grade. Discordant cases were discussed and a consensus grading was worked out. A recent study by Petersen et al. proposes a grading system for LAC based on the nuclear size variability. They could demonstrate that the core size variability of LAC tumor cells correlated significantly with the patient's survival [40]. Our data indicate that the conventional grading system provided by the WHO classification is still unsatisfactory and does not reflect the biology of the tumor. Further studies correlating different grading systems and gene expression data will be necessary to answer this question profoundly.

In our study EGFR protein expression and number of *EGFR *gene copies were analyzed by immunohistochemistry and FISH, respectively. Consistent with the literature 86% (24/28 cases) of investigated tumor samples showed an expression of EGFR on protein level [41-43]. No correlation of EGFR status and the results of gene expression profiling could be detected. A similar result was found by Balko et al. who applied a gene signature predicting the sensitivity to EGFR tyrosine kinase inhibitors obtained from various cell lines to classify human lung adenocarcinomas [6].

In contrast to previous publications, reporting an amplification frequency of approximately 10%, no amplification of the *EGFR *locus was observed in this study [41,43]. This discrepancy might in part be explained by the relative small number of LAC analyzed in this study. On the other hand our study, in contrast to other published data mainly arising from the US, originates from a homogeneous south German population, possibly reflecting genetic differences between different populations. No correlation between microarray data and TNM tumor stage, smoking status or gender was found [32,44].

In a supervised approach we performed a pathway analysis confirming an overexpression of genes involved in signal cascades downstream to EGFR. The pathway related genes showed a correlation with the histopathological tumor grade (grade 3) but not with the EGFR protein expression as determined by the standardized Dako EGFR pharm Dx™ kit for detection of non-phosphorylated EGFR, reflecting membrane protein expression but not the activation-status of EGFR. This suggests that the immunohistochemical analysis may be less sensitive than gene expression profiles to detect biologically relevant tumor characteristics linked to EGFR signaling.

Several studies have used expression profiling to characterize prognosis in lung cancer [5,6,12,16,21,22,25,26,29]. We choose the works by Balko et al., Larsen et al., Potti et al. and Cheng et al. for further analysis.

Individual unsupervised cluster analysis in the space of each of the genesets (combining all genes reported as being relevant per signature) failed to define robust clusters of cases, except the signature of 114 probe sets published by Potti et al. that resulted in some co-segregation of cases with similar outcome in our dataset [25].

GSEA analysis, used to test for enrichment of the individual prognostic gene sets confirmed significant over-representation of the signatures of Balko and Chen, and a trend towards enrichment of the Potti signature, in the top genes ranked by differential expression between long term survivors and patients with unfavorable clinical outcome, indicating an association between these gene expression signatures and the survival of the patients that remains to be characterized in larger sample sets [6,12,25].

## Conclusions

This study confirmed the limited value of published gene expression analyses to identify patients with poor outcome in a LAC dataset, as recently shown in a large multicenter study, particularly when applying it to smaller independent data sets [32]. In the light of the present data it seems unlikely, that a signature of only few mRNA measurements will be sufficient to reliably predict response/prognosis, particularly if applied to single cases or smaller series of patient samples. The gene expression signatures observed in this study seem to be mainly driven by the tumor grade, even more so than by EGFR protein expression detected by IHC and other clinical parameters. Therefore, a careful histopathological assessment and the use of consensus pathologist panels are recommended for future studies to standardize histopathological annotation and to combine gene expression signatures with robust clinical parameters.

## Competing interests

The authors declare that they have no competing interests.

## Authors' contributions

JN participated in the microarray experiments and the data analysis and drafted the manuscript. FF performed the microarray data analysis and major parts of the statistical analysis, generated the figures (except fig. 2), and contributed to the draft and final manuscript. GK performed the molecular genetic studies and the immunoassays and confirmed the histopathological diagnoses. TW carried out the microarray experiments. BP provided the clinical data and participated in the design of the study. KA participated in the statistical analysis and contributed to the final manuscript. PF participated in the design and coordination of the study. MW conceived of the study, participated in its design and coordination and confirmed the histopathological diagnoses. AZH is the principal investigator, designed and coordinated the study and drafted the manuscript. All authors read and approved the final manuscript.

## Pre-publication history

The pre-publication history for this paper can be accessed here:

http://www.biomedcentral.com/1471-2407/10/77/prepub

## Supplementary Material

Additional file 1 **Supplemental table S1.** Genes extracted from nine metagene signatures according to Potti et al.  The table contains detailed information of the Affymetrix probe set numbers, gene names and gene symbols of the 114 genes extracted from nine metagene signatures according to Potti et al. Click here for file
